# PB.1. Axillary lymph node ultrasound and needle sampling in preoperative staging of breast cancer: re-audit

**DOI:** 10.1186/bcr3711

**Published:** 2014-11-03

**Authors:** P Hamilton, H Kazi, L Clarke, A Robinson, A Leaver

**Affiliations:** 1 Gateshead Hospitals NHS Trust, Gateshead, UK; 2 South Tyneside Hospitals NHS Trust, South Tyneside, UK

## Introduction

In our unit, invasive breast cancer patients undergo axillary ultrasound and sometimes needle sampling with fine-needle aspiration or core biopsy for preoperative staging. Results triage patients to appropriate axillary surgery. We present a complete audit cycle. Between cycles 1 and 2, departmental guidelines were changed to include repeat preoperative biopsy in cases with suspicious ultrasound but negative biopsy result (LN4 and LN5 of an LN1 to LN5 node ultrasound grading system), and for patients with inconclusive biopsy results.

## Methods

The authors performed a retrospective analysis of multidisciplinary team meeting records of all invasive breast cancer patients operated upon from January 2010 to June 2010 (cycle 1), and from January 2012 to December 2013 (cycle 2). Descriptive statistics were performed.

## Results

Initial audit (cycle 1): 125 female patients with 64% (921/33) overall combined sensitivity of ultrasound/needle biopsy and 100% (92/92) specificity. Re-audit (cycle 2): 676 female patients, axillary ultrasound performed in all cases. Needle sample performed in 52.8% (357/676) patients. Repeat needle sampling in 16% (57/357) patients: 77.2% (44/57) benign, 22.8% (13/57) malignant. Ultrasound sensitivity 79.1% (163/206), needle sampling sensitivity 76.1% (124/163). Overall combined sensitivity of ultrasound/needle sampling 59% (466/469). Overall combined specificity of ultrasound/needle sampling was 99.3% (466/469).

## Conclusion

The introduction of repeat preoperative lymph node biopsy guidelines did demonstrate node metastases that were not seen on initial node biopsy, increasing accuracy of triage to an appropriate axillary surgical procedure in those patients. Unfortunately this did not lead to an improvement in overall sensitivity of preoperative axillary staging between cycles.

